# Multimodal Brain Imaging Reveals Structural Differences in Alzheimer’s Disease Polygenic Risk Carriers: A Study in Healthy Young Adults

**DOI:** 10.1016/j.biopsych.2016.02.033

**Published:** 2017-01-15

**Authors:** Sonya F. Foley, Katherine E. Tansey, Xavier Caseras, Thomas Lancaster, Tobias Bracht, Greg Parker, Jeremy Hall, Julie Williams, David E.J. Linden

**Affiliations:** a Cardiff University Medical Research Council Centre for Neuropsychiatric Genetics and Genomics, Wales, United Kingdom; b Cardiff University Brain Research Imaging Centre, School of Psychology, Wales, United Kingdom; c Central Biotechnology Services, TIME Institute, Wales, United Kingdom; d Medical Research Council Integrative Epidemiology Unit, School of Social and Community Medicine, Faculty of Medicine & Dentistry, University of Bristol, Bristol, United Kingdom; e Neuroscience and Mental Health Research Institute, Wales, United Kingdom

**Keywords:** Alzheimer’s disease, Cingulum, Fornix, Hippocampus, Imaging, Polygenic risk

## Abstract

**Background:**

Recent genome-wide association studies have identified genetic loci that jointly make a considerable contribution to risk of developing Alzheimer’s disease (AD). Because neuropathological features of AD can be present several decades before disease onset, we investigated whether effects of polygenic risk are detectable by neuroimaging in young adults. We hypothesized that higher polygenic risk scores (PRSs) for AD would be associated with reduced volume of the hippocampus and other limbic and paralimbic areas. We further hypothesized that AD PRSs would affect the microstructure of fiber tracts connecting the hippocampus with other brain areas.

**Methods:**

We analyzed the association between AD PRSs and brain imaging parameters using T1-weighted structural (*n* = 272) and diffusion-weighted scans (*n* = 197).

**Results:**

We found a significant association between AD PRSs and left hippocampal volume, with higher risk associated with lower left hippocampal volume (*p* = .001). This effect remained when the *APOE* gene was excluded (*p* = .031), suggesting that the relationship between hippocampal volume and AD is the result of multiple genetic factors and not exclusively variability in the *APOE* gene. The diffusion tensor imaging analysis revealed that fractional anisotropy of the right cingulum was inversely correlated with AD PRSs (*p* = .009). We thus show that polygenic effects of AD risk variants on brain structure can already be detected in young adults.

**Conclusions:**

This finding paves the way for further investigation of the effects of AD risk variants and may become useful for efforts to combine genotypic and phenotypic data for risk prediction and to enrich future prevention trials of AD.

Alzheimer’s disease (AD) is the most common neurodegenerative disease, affecting about 5% to 7% of the population over 60 years of age (1). Although a small proportion of cases, often with a younger onset, are caused by autosomal dominant mutations, the vast majority of cases do not follow Mendelian heritability. Such sporadic AD is mediated by both environmental and genetic factors, with many genes contributing different degrees of risk (2). The most highly penetrant common genetic risk factor for AD is the apolipoprotein E(*APOE*) ε4 allele, each copy of which increases AD risk by a factor of about 3 (3). However, recent genome-wide association studies (GWASs) have identified a further 19 genome-wide significant loci for AD (4, 5, 6), which provide new insights into possible biological mechanisms underlying the neurodegenerative process (7). Individually, the most powerful of these variants only marginally increase an individual’s risk for developing AD (~1% to 8%) (4). Polygenic risk scores (PRSs), which are based on the additive effect of multiple loci across the genome, may be better suited to capture the variance explained by common alleles (8). PRSs based on the most recent GWASs have considerable predictive utility for AD risk (9).

Structural brain imaging has consistently revealed both global and local atrophic changes in patients with AD (10) and is a useful biomarker for preclinical disease (11). Local atrophy in medial temporal areas, including the hippocampus, is observed early in the course of the disorder (12, 13) and in patients with mild cognitive impairment (MCI) (14), a clinical state that may be a precursor to AD (15). Hippocampal atrophy predicts conversion from MCI to AD (16) and has also been reported in carriers of rare dominant AD risk variants in the genes coding for amyloid precursor protein and presenilin 1 (17), as well as carriers of highly penetrant common variants such as *APOE* ε4 (18). Indeed, hippocampal volume is already a key imaging phenotype to identify preclinical stages of AD (19). Other brain structures showing significant atrophy early in disease progression include other medial temporal lobe regions including the entorhinal cortex (ERC) (20, 21), parahippocampal gyrus (PHG) (22), and posterior cingulate gyrus (PCG) (23). We therefore focused on early changes in these structures.

The combination of PRSs and neuroimaging data is likely to be particularly informative in identifying markers of early risk for AD (24, 25), even before the putative onset of amyloid accumulation. In the current study, we sought to investigate the correlation between polygenic risk for AD based on the largest genetic training dataset available (4) and gray and white matter structural differences in a healthy young population without any signs of cognitive impairment. So far, the only studies conducted with AD PRSs have used 24 risk loci (26) or PRSs derived from a smaller AD GWASs (23, 27), making ours the genetically most powerful study conducted on this topic to date.

We predicted that the PRSs for AD would be negatively correlated with hippocampal volume. We were also interested in exploring the association of cortical thickness of ERC, PHG, and PCG, due to the involvement of these areas in early AD (28, 29, 30), where we would expect to see a decrease in thickness. In addition to gray matter parameters, we also investigated the microstructure of the main white matter pathways connecting our candidate areas to ascertain whether any gray matter loss would already have impacted on the fiber tracts by way of anterograde or retrograde degeneration. We measured fractional anisotropy (FA), a measure of white matter microstructure derived from diffusion tensor imaging (DTI) (31) in the main connecting tracts of the hippocampus, the cingulum, and the fornix. We expected FA to be lower in participants with higher AD PRSs.

## Methods and Materials

### Participants

Brain scans used in this study were obtained from a repository of neuroimaging and genetic data obtained between 2009 and 2014 from healthy subjects recruited through a range of research projects at Cardiff University Brain Research Imaging Centre, which has received ethical approval from the Cardiff University School of Psychology. All subjects were screened for the exclusion of any neuropsychiatric disorders either by interview or by questionnaires. Participants provided informed consent for genotyping and use of their imaging data for genetic imaging analysis. After genotyping and data quality control standards, 272 individuals with structural T1 data remained (195 female, 77 male) with an average age at time of inclusion of 24.8 years (SD 6.9). Tractography data were available for a subset of 197 participants (138 female, 59 male) with an average age at time of inclusion of 23.9 years (SD 5.1). For a subgroup of 87 participants (53 female, 34 male; mean age 23.9 years [SD 4.4]), data on the Hopkins Verbal Learning Task were available. This task measures declarative verbal learning capacity (32) and forms part of the MATRICS Consensus Cognitive Battery.

### Genotyping

Genomic DNA was obtained from saliva using Oragene OG-500 saliva kits (DNA Genotek, Inc., Ontario, Canada). Genotyping was performed using custom Illumina HumanCoreExome-24 BeadChip genotyping arrays, which contain 570,038 genetic variants (Illumina, Inc., San Diego, CA). Quality control was implemented in PLINK (33). Individuals were excluded for any of the following reasons: 1) ambiguous sex (genotypic sex and phenotypic sex not aligning); 2) cryptic relatedness up to third-degree relatives as ascertained using identity by descent; 3) genotyping completeness less than 97%; and 4) non-European ethnicity admixture. The latter was detected as outliers in an iterative EIGENSTRAT analysis of a linkage-disequilibrium-pruned dataset (34). Single nucleotide polymorphisms (SNPs) were excluded where the minor allele frequency was less than 1%, if the call rate was less than 98%, or if the χ^2^ test for Hardy-Weinberg equilibrium had a *p* value less than 1 × 10^−4^. Individuals’ genotypes were imputed using the prephasing/imputation stepwise approach implemented in IMPUTE2/SHAPEIT (35, 36) and 1000 Genomes (December 2013, release 1000 Genomes haplotypes Phase I integrated variant set) (37) as the reference dataset. This resulted in a dataset of 274 individuals with information for 7,413,342 SNPs.

### Polygenic Scoring Method

Polygenic score calculations were performed according to the procedure described by the International Schizophrenia Consortium (38). Training data were from the International Genomics of Alzheimer’s Project consortium that comprises 17,008 AD cases and 37,154 control subjects (4). These data are publicly available from http://www.pasteur-lille.fr/en/recherche/u744/igap/igap_download.php. SNPs were removed from all analyses if they had a low minor allele frequency (< .01). Subsequently, the data were pruned for linkage disequilibrium using the clumping function (--clump) in PLINK (33) removing SNPs within 500 kilobase (--clump-kb) and r^2^ > .25 (--clump-r2) with a more significantly associated SNP. We used the --score command in PLINK to calculate polygenic scores (33). Nine different progressive training *p* value thresholds (39) were investigated (polygenic threshold [P_T_] < 1 × 10^−8^, 1 × 10^−7^, 1 × 10^−6^, 1 × 10^−5^, 1 × 10^−4^, .01, .1, .3, and .5). Lower P_T_ indicates that SNPs are more significantly associated with AD case status in the training dataset (AD case-control study) (4).

These polygenic risk scores include the *APOE* loci on chromosome 19, the greatest common genetic risk factor for AD. If a significant association was observed between AD PRSs and brain imaging phenotypes, the data were reanalyzed with polygenic scores excluding any SNPs within the *APOE* locus (chromosome 19: 45.053–45.73 Mb), to assess if the association was purely due to variance in *APOE*.

### Magnetic Resonance Imaging Data

#### Data Acquisition

Magnetic resonance imaging was carried out in Cardiff University Brain Research Imaging Centre on a GE Signa HDx 3T scanner (GE Healthcare, Milwaukee, WI). T1-weighted structural data were acquired using an axial three-dimensional fast, spoiled gradient recalled sequence with the following parameters: repetition time/echo time/inversion time = 8/3/450 ms; flip angle = 20°; 1 mm resolution; field of view ranging from 256 × 192 × 160 mm^3^ to 256 × 256 × 256 mm^3^ (anterior–posterior/left–right/superior–inferior), with acquisition time ranging from approximately 6 minutes to 10 minutes.

DTI data were acquired using a cardiac-gated sequence with the following parameters; b-values 0 and 1200, repetition time ~20 seconds (dependent on heart rate); echo time = 90 ms; 60 2.4-mm slices aligned with the anterior commissure-posterior commissure, zero slice gap; acquisition matrix 96 × 96; field of view = 230 mm; 2.4-mm isotropic resolution. Data were either acquired from 30 unique diffusion directions plus 3 b0 images or from a subsample of 30 optimal directions from an acquired set of 60 directions with the first 3 b0 images.

#### Data Processing

Hippocampal volume, ERC, PHG, PCG thickness, and intracranial volume (ICV) were determined through analysis with FreeSurfer (surfer.nmr.mgh.harvard.edu), which has been validated as a suitable method for hippocampal segmentation in large samples (40). The resulting output was quality controlled following a publically available protocol from ENIGMA (http://enigma.ini.usc.edu/) (41). Whenever a region of interest was detected as inadequately segmented by XC or SFF, its metric was declared missing and excluded from analysis. The numbers of brain regions included in the final analysis were hippocampus right and left (*n* = 270), entorhinal cortex left (*n* = 257) and right (*n* = 268), posterior cingulate gyrus left (*n* = 272) and right (*n* = 271), and parahippocampal gyrus left (*n* = 259) and right (*n* = 271).

DTI data were analyzed using ExploreDTI (42) version 4.8.3 and were corrected for eddy current distortions and subject motion using an affine registration to the nondiffusion-weighted images, with appropriate reorienting of the encoding vectors (43). An echo planar imaging (4) correction was applied, warping the DTI data to the fast, spoiled gradient recalled images, resulting in a 1 × 1 × 1 mm^3^ resolution in the resulting output. A single diffusion tensor model was fitted to the DTI data (44) to compute quantitative parameters such as FA.

**Figure 1:**
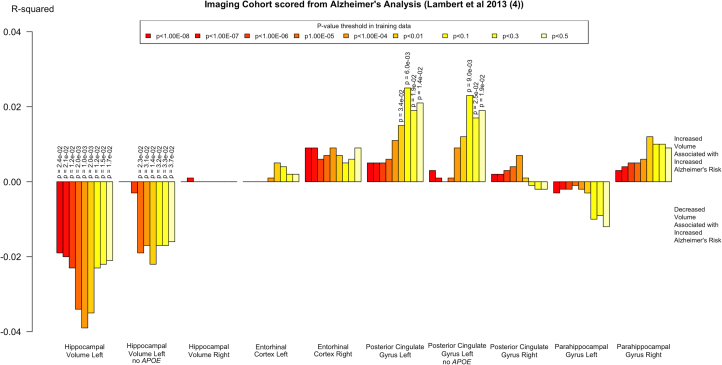
**(A)** Coronal view of fornix fibers. **(B)** Sagittal view of right cingulum fibers. **(C)** Sagittal view of right parahippocampal cingulum fibers.

Subsequently, the damped Richardson Lucy pipeline (45) was used to perform whole-brain tractography. Termination criteria were an angle threshold greater than 45° or a drop in the magnitude of the minimally subtending fiber orientation density function peak below 0.05. Tracts were obtained using in-house automated tractography software (46). The automated tractography models for the fornix, cingulum, and parahippocampal cingulum (PHC) were based on manual tractography performed by SFF. Each automated tract underwent quality control through visual inspection and was brought to manual tractography standards by post hoc removal of any fiber bundles considered spurious, where necessary. Final numbers of tracts included were fornix *n* = 157 and cingulum and PHC right and left *n* = 197. The fornix was defined according to Metzler-Baddeley *et al.* (47); the high levels of dropout were most probably due to the high curvature of the tract and proximity to the ventricles. To segment the PHC, the restricted method was used, which incorporates a NOT gate-blocking inclusion of all tracts projecting toward the frontal cortex (48). For an example of the tracts, see Figure 1. Free water correction was applied (49, 50) before extracting FA values for further analysis. FA values were extracted using customized MATLAB scripts (The MathWorks, Inc., Natick, MA).

#### Analysis

Regional thickness data determined from T1 scans were analyzed using hierarchical linear multiple regression in IBM SPSS statistics 20 (IBM Corp., Armonk, NY), covarying for age, gender, and ICV. The hippocampal volumes were adjusted for ICV of each participant with the formula: hippocampal volume corrected = hippocampal volume − [beta * (ICV − mean ICV across the group studied)] (51). Subsequently, they were analyzed using hierarchical linear multiple regression covarying for age and gender.

The DTI data were also analyzed using hierarchical linear multiple regression, including FA as the dependent variable and controlling for independent variables age, gender, and scan type (30 or downsampled 60 directions).

The *p* values were then corrected for multiple comparisons using false discovery rate in statistics package R (52), resulting in *q* values. False discovery rate was applied over the regions of interest studied. Where significant results remained after false discovery rate correction, further analysis was performed using the PRSs without *APOE* SNPs.

It is standard practice to compare PRSs over multiple thresholds (38), and it is difficult to correct for multiple comparisons due to the highly correlated nature of the thresholds. Permutation testing is a robust way to correct for multiple comparisons in a dependent sample (53). The Supplement contains an outline of permutation tests performed on all the nominally significant results.

## Results

### Hippocampal Volume

PRSs correlated negatively with left hippocampal volume corrected for intracranial volume (*R*^2^ = .039; *p* = .001; *q* = 0.008; P_T_ < 1 × 10^−4^) as demonstrated in Table 1. PRSs calculated at all *P*_T_s were nominally associated with decreased left hippocampal volume, and four of these survived correction for multiple testing (Table 1). Analysis of the *R*^2^ change showed that the PRSs accounted for an additional 1.9% to 3.9% of the variance in left hippocampal volume over the nine thresholds, after removing variance explained by age and gender, which accounted for 0.5%. No such effect was seen in the right hippocampus, where *R*^2^ changed ≤.001 across the whole range of PRS thresholds.

**Figure 2:**
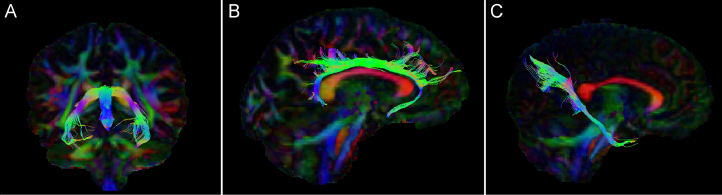
Effects of polygenic risk scores on regions of interest before false discovery rate correction. Vertical axis denotes the *R*^2^ change, with upward indicating an increase and downward indicating a decrease of volume or thickness of the region of interest.

Subsequent analysis of hippocampal volume with the *APOE* locus removed from PRSs showed that the significant association between AD PRSs and decreased left hippocampal volume persisted (*R*^2^ = .022; *p* = .014; P_T_ < .01), particularly with the more inclusive *p*_T_s (Figure 2).

### Entorhinal Cortex, Posterior Cingulate Gyrus, and Parahippocampal Gyrus Thickness

No significant effects of AD PRSs were seen on the volume of the ERC or PHG. A small increase in volume was seen in the left PCG, which was conserved at one threshold (*R*^2^ = .025; *p* = .006; *q* = 0.048; P_T_ < .1), after correction for multiple comparisons. This effect persisted after removal of the *APOE* locus from the PRSs (Table 1).

### Tractography Fornix, Cingulum, and PHC

No effect of AD PRSs on FA was found in the fornix or PHC (Table 2). However, a significant effect of AD PRSs on FA of the right cingulum was found (*R*^2^ = .032; *p* = .009; *q* = 0.045; P_T_ < 1 × 10^−4^), with a negative association between PRS and FA at five thresholds. This effect was conserved for one of these thresholds (*p* ≤ .05) when correcting for multiple comparisons over all five regions of interest. Subsequent analysis of cingulum FA, excluding the *APOE* locus from the AD PRSs, only showed a significant association at one threshold (*R*^2^ = .019; *p* = .044; P_T_ < 1 × 10^−6^).

### Cognitive Effects

We found no correlation between scores on the Hopkins Verbal Learning Task and polygenic risk for AD (Supplemental Table S1).

## Discussion

As predicted, PRS for AD was associated with lower hippocampal volume. While hippocampal volume reductions in patients are a robust finding in AD (10, 13) and MCI patients converting to AD undergo greater hippocampal atrophy (16), previous reports of hippocampal volume effects of genetic risk variants in healthy subjects have been varied. Some studies (54, 55, 56) found no effect of *APOE* risk alleles on hippocampal volume in young participants, while others found a significant difference between young ε4 and ε2 carriers (18). Previously, right, but not left, hippocampal volume reductions were found in *APOE* ε4 risk allele carriers in comparison with ε3 carriers in a middle-aged population (57), whereas we found effects in the left, but not right, hippocampus. However, most of the previous literature has reported a slight preponderance of left compared with right hippocampal changes, particularly in the preclinical and early stages of AD progression (58), which would be in line with our findings.

We may see significant effects where others have obtained mixed results with single locus studies because of the benefits of using polygenic risk scores. Although the SNPs contributing to the PRSs have much smaller individual effect sizes than the *APOE* locus, cumulatively they explain a large amount of variance (9).

Although previous reports have implicated the ERC in AD pathology (20, 21), we do not see an effect of AD genetic risk on ERC thickness. This is consistent with previous studies (39) that failed to find an effect of APOE on ERC thickness in a healthy middle-aged and older adult population. Conversely, the small increase in PCG thickness was unexpected because both manifest AD and genetic risk status have been associated with thinning of this area (59, 60). Replication of this finding in future studies would be needed before strong conclusions can be drawn.

It has been proposed that AD, like many other neuropsychiatric disorders, is a disconnection syndrome (61). Connectivity models of pathophysiology can be supported by the investigation of the microstructural properties of white matter. Changes in white matter parameters have indeed been observed in many DTI studies of AD, for example, decreased FA in the right fornix (10) and the left PHC (62), superior longitudinal fasciculus, temporal lobe (63, 64), and cingulum (65). A significant decrease of FA was also found in the PHC in MCI patients (66). Differences in FA relating to AD risk genes have previously been observed in healthy participants as well; *APOE* ε4 was linked to alterations in FA in the left medial temporal lobe and in the corpus callosum in healthy individuals (67). One study found widespread FA decreases in a young healthy population (age 20–35) with *APOE* risk variants (68), while another found widespread decreases in FA, including in the fornix and cingulum, in young individuals carrying the clusterin risk allele (69). A population of preclinical subjects with an autosomal dominant AD variant also had significantly lower FA in the columns of the fornix (70).

Our finding of reduced FA in the right cingulum, which was not exclusively driven by the *APOE* locus, is in line with models that assume early white matter changes in the course of the development of AD pathology. The fact that this correlation between cingulum FA and AD PRSs was not driven by the PHC, which connects the medial temporal lobe with areas in the parietal and occipital lobes such as the posterior cingulate cortex (47), was contrary to expectations. In our sample, the white matter findings were not directly related to the gray matter structural differences that occurred in the opposite hemisphere.

We used the results from the largest AD GWASs undertaken to date as our training data (4). Therefore, the estimates of SNP effects on AD risk utilized in this study are the best reported to date with better power than previous estimates, as it is known that polygenic risk score and *R*^2^ values are highly affected by the sample size of the training dataset (71).

Hippocampal volume and cortical thickness are highly heritable, which makes them appropriate parameters for genetic imaging analysis (72, 73). A limitation of our study is that, by the nature of the polygenic analysis, which pools risk variants across the whole genome, no inferences can be made on the specific molecular mechanisms contributing to the structural brain differences. Although neurofibrillary tangles can be present in the hippocampus in young adults, the Braak staging model would suggest that entorhinal/transentorhinal cortex is affected even earlier by this process (74, 75). Although amyloid pathology can be detected in young carriers of Mendelian variants affecting the amyloid pathway (76), it would be unlikely to be confined to the hippocampus (75), and our sample was probably too young to have a significant amyloid load (77). GWASs for AD have revealed novel pathways associated with AD, including in lipid metabolism, immune responses, and endocytosis, while also finding no enrichment of genes associated with either tau or amyloid pathways, suggesting other factors may play a role in risk and development of AD (7). Furthermore, it is possible that the cumulative effect of common AD risk variants affects the development of the hippocampus in a similar way, as they have been shown to affect cognition across the life course of an individual (78). Longitudinal studies of genetic imaging, involving even younger populations than that of the present study, and pathway-based analysis (79, 80, 81) will be needed to address the biological significance of our findings. However, pathway-based analyses will likely require larger samples and new approaches to the multiple testing problem.

In conclusion, AD polygenic risk was associated with smaller volume of the left hippocampus, increased volume of left PCG, and lower FA in the right cingulum bundle in healthy young adults. Thus, AD genetic risk can be linked to structural differences in brain areas that have been implicated in the early stages of AD pathology many decades before potential illness onset. This effect was not driven exclusively by contributions from *APOE,* as the associations persisted after removal of the *APOE* locus. Overall, this work suggests that genetic risk for AD is mediated, in part, through brain morphological differences, mainly in the hippocampus, confirming hippocampal volume changes as an important early biomarker of risk for AD.

## Figures and Tables

**Table 1:** The Influence of Polygenic AD Risk Scores on Brain Structure

Training P_T_ Value	Hippo-campus L *R*^2^ (*p* Value)	Hippo-campus L No *APOE R*^2^ (*p* Value)	Hippo-campus R *R*^2^ (*p* Value)	ERC L *R*^2^ (*p* Value)	ERC R *R*^2^ (*p* Value)	PCG L *R*^2^ (*p* Value)	PCG L No *APOE R*^2^ (*p* Value)	PCG R *R*^2^ (*p* Value)	PHG L *R*^2^ (*p* Value)	PHG R *R*^2^ (*p* Value)
P_T_ < 1 × 10^−8^	.019 (.024)^a^	<.001 (.966)	<.001 (.795)	<.001 (.742)	.009 (.118)	.005 (.224)	.003 (.391)	.002 (.500)	.003 (.386)	.003 (.393)
P_T_ < 1 × 10^−7^	.020 (.021)^a^	<.001 (.991)	.001 (.683)	<.001 (.869)	.009 (.127)	.005 (.213)	.001 (.550)	.002 (.486)	.002 (.433)	.004 (.325)
P_T_ < 1 × 10^−6^	.023 (.012)^a^	.003 (.409)	<.001 (.814)	<.001 (.833)	.006 (.215)	.005 (.215)	<.001 (.788)	.003 (.362)	.002 (.522)	.005 (.227)
P_T_ < 1 × 10^−5^	.034 (.002)^a^^,^^b^	.019 (.023)^a^	<.001 (.958)	<.001 (.873)	.007 (.173)	.006 (.201)	.001 (.635)	.004 (.287)	.001 (.592)	.005 (.256)
P_T_ < 1 × 10^−4^	.039 (.001)^a^^,^^b^	.017 (.031)^a^	<.001 (.932)	.001 (.620)	.009 (.128)	.011 (.073)	.009 (.112)	.007 (.156)	.002 (.481)	.006 (.212)
P_T_ < .01	.035 (.002)^a^^,^^b^	.022 (.014)^a^	<.001 (.933)	.005 (.257)	.007 (.181)	.015 (.034)^a^	.012 (.060)	.001 (.554)	.003 (.406)	.012 (.076)
P_T_ < .1	.023 (.012)^a^^,^^b^	.017 (.032)^a^	<.001 (.863)	.004 (.289)	.005 (.253)	.025 (.006)^a^^,^^b^	.023 (.009)^a^	.001 (.645)	.010 (.109)	.010 (.099)
P_T_ < .3	.022 (.015)^a^	.017 (.033)^a^	<.001 (.850)	.002 (.512)	.006 (.206)	.019 (.019)^a^	.017 (.025)^a^	.002 (.488)	.009 (.123)	.010 (.094)
P_T_ < .5	.021 (.017)^a^	.016 (.037)^a^	<.001 (.748)	.002 (.530)	.009 (.123)	.021 (.014)^a^	.019 (.019)^a^	.002 (.483)	.012 (.082)	.009 (.128)

*R*^2^ change and *p* values for hippocampal volume (left *n* = 270; right *n* = 270), entorhinal cortex thickness (left *n* = 257; right *n* = 268), posterior cingulate gyrus thickness (left *n* = 272; right *n* = 271), and parahippocampal gyrus thickness (left *n* = 259; right *n* = 271). The top axis shows the region of interest, while the vertical axis shows the polygenic threshold.

AD, Alzheimer’s disease; ERC, entorhinal cortex; FDR, false discovery rate; L, left; PCG, posterior cingulate gyrus; PHG, parahippocampal gyrus; P_T_, polygenic threshold; R, right.

a Nominally significant associations (*p* value < .05).

b Denotes q-FDR corrected <0.05.

**Table 2:** *R*^2^ Change and *p* Values for the Change in FA Correlating With AD PRS for Each Threshold

Training P_T_ Value	Fornix *R*^2^ (*p* Value)	Cingulum L *R*^2^ (*p* Value)	Cingulum R *R*^2^ (*p* Value)	Cingulum R No *APOER*^2^ (*p* Value)	PHC L *R*^2^ (*p* Value)	PHC R *R*^2^ (*p* Value)
P_T_ < 1 × 10^−8^	<.001 (.791)	.009 (.164)	.030 (.011)^a^	.006 (.254)	.008 (.194)	.002 (.534)
P_T_ < 1 × 10^−7^	<.001 (.813)	.009 (.162)	.029 (.013)^a^	.006 (.268)	.006 (.233)	.002 (.515)
P_T_ < 1 × 10^−6^	<.001 (.813)	.005 (.270)	.023 (.026)^a^	.019 (.044)^a^	.006 (.255)	.002 (.538)
P_T_ < 1 × 10^−5^	<.001 (.846)	.004 (.344)	.025 (.020)^a^	.006 (.270)	.005 (.292)	.001 (.655)
P_T_ < 1 × 10^−4^	<.001 (.780)	.009 (.165)	.032 (.009)^a^^,^^b^	<.001 (.885)	.002 (.547)	<.001 (.949)
P_T_ < .01	<.001 (.952)	.001 (.610)	.006 (.273)	<.001 (.858)	.003 (.432)	.001 (.727)
P_T_ < .1	<.001 (.886)	.001 (.626)	.005 (.305)	.001 (.588)	.005 (.289)	.006 (.268)
P_T_ < .3	<.001 (.840)	.003 (.440)	.002 (.493)	<.001 (.777)	.003 (.388)	.004 (.357)
P_T_ < .5	<.001 (.806)	.002 (.486)	.002 (.471)	.001 (.731)	.003 (.387)	.007 (.240)

Fornix (*n* = 157), cingulum left (*n* = 197) and right (*n* = 197) (with and without *APOE* SNPs), and parahippocampal cingulum left (*n* = 197) and right (*n* = 197) are shown here.

AD, Alzheimer’s disease; FA, fractional anisotropy; FDR, false discovery rate; L, left; PRS, polygenic risk score; PHC, parahippocampal cingulum; P_T_, polygenic threshold; R, right; SNP, single nucleotide polymorphism.

a Nominally significant associations (*p* value < .05).

b Associations that survive FDR correction.

## Footnotes

Appendix A Supplementary material cited in this article is available online at 10.1016/j.biopsych.2016.02.033.
