# Supplementary material for: Multimodal Brain Imaging Reveals Structural Differences in Alzheimer’s Disease Polygenic Risk Carriers: A Study in Healthy Young Adults
Source: Biol Psychiatry. 2017 Jan 15;81(2):154–61. doi: 10.1016/j.biopsych.2016.02.033 (PMC5177726; doi:10.1016/j.biopsych.2016.02.033)
Supplement: Supplementary file 1 — Supplementary material [file mmc1.pdf]

Multimodal Brain Imaging Reveals Structural Differences in Alzheimer's Disease Polygenic Risk Carriers: A study in Healthy Young Adults

Supplemental Information

Table S1. Hopkins Verbal Learning Task correlation with PRS in *n* = 87 subsample

Training *P*-Value Threshold	HVLT R^2 (*P*-Value)
$P_T < 1 \times 10^{-8}$	0.006 (0.468)
$P_T < 1 \times 10^{-7}$	0.005 (0.522)
$P_T < 1 \times 10^{-6}$	0.004 (0.539)
$P_T < 1 \times 10^{-5}$	0.003 (0.587)
$P_T < 1 \times 10^{-4}$	0.001 (0.743)
$P_T < 0.01$	0.000 (0.890)
$P_T < 0.1$	0.000 (0.980)
$P_T < 0.3$	0.001 (0.775)
$P_T < 0.5$	0.001 (0.753)

Permuted *P*-Values

As a post hoc correction for multiple comparisons, we calculated permutation-based *p*-values to assure the associations found were robust. We randomly shuffled the phenotype within individuals, breaking the link between polygenic score and phenotype, and performed a linear regression on the reshuffled data set. This process was repeated 10,000 times to generate an empirical distribution of *p*-values. We calculated the permuted *p*-value as the proportion of times the *p*-values in the permuted data sets were at least as significant as the original association. The table below shows the permuted *p*-values, which all retain their significance.

Table S2. Permutation-based *p*-values of significant results

Training *P*-value Threshold	Hippocampus L R^2 (*P*-Value)	Hippocampus L No *APOE* R^2 (*P*-Value)	PCG L R^2 (*P*-Value)	PCG L No *APOE* R^2 (*P*-Value)	Cingulum R R^2 (*P*-Value)	Cingulum R No *APOE* R^2 (*P*-Value)
$P_T < 1 \times 10^{-8}$	0.0244	NA	NA	NA	0.0122	NA
$P_T < 1 \times 10^{-7}$	0.0204	NA	NA	NA	0.0112	NA
$P_T < 1 \times 10^{-6}$	0.0113	NA	NA	NA	0.026	0.0438
$P_T < 1 \times 10^{-5}$	0.0021	0.0236	NA	NA	0.0207	NA
$P_T < 1 \times 10^{-4}$	0.0011	0.032	NA	NA	0.0074	NA
$P_T < 0.01$	0.0028	0.0137	0.0337	NA	NA	NA
$P_T < 0.1$	0.011	0.0276	0.0052	0.0092	NA	NA
$P_T < 0.3$	0.0159	0.0338	0.0175	0.0239	NA	NA
$P_T < 0.5$	0.0177	0.038	0.0124	0.0184	NA	NA

Correlation Between Right and Left Hemisphere ROIs

As right and left hemisphere volumes are usually highly correlated, but our effects were all lateralized, suggestions were made to analyze bilateral hemisphere correlations for volume and thickness.

Bivariate correlations were performed. Hippocampal volume and ROI thicknesses were highly correlated between hemispheres:

Left hippocampus – Right hippocampus: Pearson correlation 0.448 ($p < 0.001$)

Left ERC – Right ERC: Pearson correlation 0.350 ($p < 0.001$)

Left PCG – Right PCG: Pearson correlation 0.564 ($p < 0.001$)

Left PHG – Right PHG: Pearson correlation 0.510 ($p < 0.001$)

Linear multiple regression was performed of genetic AD risk scores with the mean volume or thickness of bilateral ROIs, The results can be seen in Table S3.

Table S3. Linear regression of bilateral brain regions with PRS. As seen here, the effects previously found were mostly lost, with the exception of three thresholds for hippocampal volume. Considering our previous results, we would suggest it shows that effects in the left hemisphere were strong enough to survive even if we had looked at averaged right and left hemisphere.

P-Value Threshold	Hippocampus R^2 (*P*-Value)	ERC R^2 (*P*-Value)	PCG R^2 (*P*-Value)	PHG R^2 (*P*-Value)
$P_T < 1 \times 10^{-8}$	0.005 (0.144)	0.003 (0.345)	0.004 (0.290)	0.000 (0.871)
$P_T < 1 \times 10^{-7}$	0.005 (0.154)	0.004 (0.308)	0.004 (0.276)	0.000 (0.969)
$P_T < 1 \times 10^{-6}$	0.007 (0.098)	0.002 (0.426)	0.005 (0.224)	0.000 (0.873)
$P_T < 1 \times 10^{-5}$	*0.011 (0.032)*	0.005 (0.281)	0.006 (0.184)	0.000 (0.842)
$P_T < 1 \times 10^{-4}$	*0.012 (0.024)*	0.008 (0.158)	0.011 (0.068)	0.000 (0.893)
$P_T < 0.01$	*0.011 (0.034)*	0.010 (0.117)	0.007 (0.135)	0.001 (0.636)
$P_T < 0.1$	0.009 (0.058)	0.008 (0.148)	0.005 (0.234)	0.000 (0.967)
$P_T < 0.3$	0.006 (0.121)	0.007 (0.180)	0.002 (0.404)	0.000 (0.924)
$P_T < 0.5$	0.005 (0.147)	0.009 (0.140)	0.003 (0.372)	0.000 (0.884)
